# Letter from Portland, Ore.

**Published:** 1905-05

**Authors:** William House

**Affiliations:** Class of ’95, U. of B.; Portland, Oregon


### CORRESPONDENCE.

### Letter from Portland, Ore.

**The American Medical Association at Portland—The City and its Environment—Hotel Accommodations for the Visitors—The Lewis & Clark Exposition—Dr. House and His Colleagues—University of Buffalo Graduates in the Northwest.**

*Editor Buffalo Medical Journal:*

Sir—The approaching meeting of the A. M. A. in Portland, Oregon, July 11-15, may lend local interest to a letter concerning that meeting written by a former Buffalonian, who for the past five and one-half years has resided in or near Portland. If I need any further apology for intruding upon your space, I have it in the shape of numerous letters of inquiry concerning the states of Oregon and Washington from graduates of the medical college, and residents of the city of Buffalo.

It is to be hoped that every physician who can possibly get away will take advantage of the unusual opportunity to visit the northwest, afforded by the approaching session of this representative body of medical men. Few there are, able to appreciate the conditions of a country so far from home without first visiting it, and I am certain that all who come to Portland will go away well pleased with their visit. Leaving out all, save mere mention of the scenic wonders to be enjoyed enroute, these two states will certainly prove a revelation to the beholder who, for the first time, enters their portals. Both Oregon and Washington are growing rapidly, new towns are springing up all over both states, and old ones enlarging in a surprising manner. On a visit, one must not fail to see Spokane, Seattle, and Tacoma, either coming or going, and enjoy their western hospitality. But in Portland, particularly, will most be found to repay the time and money expended on the long trip.

Primarily, Portland is a city which, since 1890, has added nearly one hundred thousand people to the population of fifty thousand, or thereabout, at that time recorded by the United States census. It is situated about six miles from the Columbia on the Willamette river, a stream capable of giving passage to the largest of the transpacific vessels and accordingly forming a harbor of no mean value. And, by the way, if you do not wish to be regarded as a tenderfoot, by all means, pronounce it Willamétte, with the accent on the second syllable. Portland partakes more of the generally accepted character of eastern cities than its immediate neighbors, and is regarded as a conservative town, a term highly complimentary to my mind as, associated with the rapidly increasing population, it indicates stability in business and social life. The town is a good one in every sense of the word.

The American Medical Association will, I think, be well entertained. As an evidence of the generosity and character of the local medical profession, I am informed that within two hours after the financial committee began to ask for subscriptions to pay expenses, twenty-five hundred dollars was subscribed in lots of five hundred dollars each, from five out of the first six men approached. These men are, of course, among the leaders, but others have been liberal according to their means and it cannot be doubted that a very considerable sum will be raised for the entertainment of visitors. Hotel accommodations are being secured by a committee headed by our best known boniface, Mr. H. C. Bowers, of the Portland Hotel, and as new hotels are springing up like mushrooms, it seems assured that they will be ample so that no one need fear inability to secure rooms, especially if reservations are made in advance. Ample space for session meetings also seems assured.

By no means least amongst the attractions will be the Lewis and Clark Exposition. This has been compared to the Buffalo Exposition by hundreds of recent visitors to the grounds, for its scope is similar to that in size and purpose. Not so large as the

Saint Louis Exposition, it is far better arranged and visitors will find it prepared on beauty lines superior to any of the previous world's fairs. In two years a veritable fairyland has sprung up, putting to shame the tales of Aladdin's lamp. Compact, orderly, and beautiful in every detail, it would prove a credit to a far older community, and those who have the double opportunity of visiting both it and the American Medical Association meeting will regret it if they fail to come.

I have been so often asked in letters as to matters medical in the northwest that, for the benefit of those seeking locations, I would like to state a few facts concerning Oregon and Washington. The number of doctors averages about one to every four hundred and seventy population. This, of course, does not include the unregistered and irregular practitioners. Among the men are many able ones in every branch. The country towns are filled, in many instances, with young easterners who tarry for awhile, and then with eager and ambitious ideals, flock to the cities. Spokane and Seattle, particularly, impress one as being overfilled with men, all young, energetic and for the most part able. Portland, no less well supplied, has perhaps fewer young easterners and this is due to the fact that we have a medical college in this city, which turns out annually from twenty to thirty graduates, while the neighboring town of Salem adds another dozen or so from its school, and usually after postgraduate work in the east, these men return to fill every available opening. Notwithstanding all this, it seems that many men achieve success in these states, though I am sure that, except in the matter of somewhat better fees, the chances are not superior to those in the east.

Among the eastern men residing in the northwest, by an odd occurrence, there are three from my class, who alphabetically responded to class call in order in which they afterward came west: Heussy, House, and Howells. Heussy has achieved distinguished success as a general practitioner with special leaning towards obstetrics, and enjoys one of the best family practices in Seattle. Howells, as medical superintendent of the Eastern Washington Hospital for the Insane, near Spokane, is also not unknown to fame, while I occupy a somewhat humbler position as resident physician in a growing and prosperous private sanitorium for nervous and insane cases, medically managed by Coe, Gillespie, and House, which in the fulness of time, with the progression of my somewhat older colleagues to satiety in matters medical, I hope to change to House & Company.

Portland and Portland doctors, with me, hope to see many Buffalonians this summer, to all of whom a true "Western Welcome" will be accorded.

WILLIAM HOUSE,
Class of '95, U. of B.

Portland, Oregon, April 17, 1905.

